# Effects of an educational intervention to strengthen humanistic practice on haemodialysis nurses’ caring attitudes and behaviours and quality of working life: a cluster randomised controlled trial

**DOI:** 10.1186/s12912-021-00729-6

**Published:** 2021-12-20

**Authors:** Matteo Antonini, Tanja Bellier-Teichmann, Louise O’reilly, Chantal Cara, Sylvain Brousseau, Jean Weidmann, Delphine Roulet-Schwab, Isabelle Ledoux, Mario Konishi, Jérôme Pasquier, Philippe Delmas

**Affiliations:** 1 La Source, School of Nursing, HES-SO, University of Applied Sciences and Arts Western Switzerland, Delémont, Switzerland; 2 Université de Montréal, Montréal, Canada; 3 Université du Québec en Outaouais, Gatineau, Canada; 4 School of Management and Engineering Vaud, Yverdon-les-Bains, Switzerland; 5 Université de Sherbrooke, Sherbrooke, Canada; 6 Institute of Social and Preventive Medicine, University Hospital of Lausanne, Lausanne, Switzerland

**Keywords:** Watson’s theory of human caring, Haemodialysis, Quality of working life, Nurse-patient relationship, Educational intervention

## Abstract

**Background:**

Nurses are trained to establish a trusting relationship with patients to create an environment promoting patients’ quality of life. However, in tech-heavy care settings, such as haemodialysis units, dehumanising practices may emerge and take root for various reasons to the potential detriment of both patients and nurses. For patients, this may lead to a deterioration of quality of life and, ultimately, of health status. For nurses, it may cause a deterioration of the work environment and, in turn, of quality of working life. Based on Watson’s Theory of Human Caring, we developed a brief educational intervention for haemodialysis nurses to strengthen their humanistic practice in the aim of improving the nurse-patient relationship and nurse quality of working life.. The intervention was tested by way of an experimental design.

**Methods:**

One hundred and one haemodialysis nurses, recruited in ten hospitals in French-speaking Switzerland, were randomised into an experimental group that received the intervention and a control group. The nurse-patient relationship was measured with the Caring Nurse-Patient Interaction Scale (EIIP-70) and nurse quality of life at work was measured with the Quality of Work Life Questionnaire at four time points: pre-intervention, intervention completion, and six-month and one-year follow-ups. Random intercept regression analysis was used to evaluate change over time in the two variables under study.

**Results:**

The intervention appeared to reinforce nurse attitudes and behaviours geared to a more humanistic practice. The effect seemed to fade over time but, 1 year post-intervention, six dimensions of the nurse-patient relationship (hope, sensibility, helping relationship, expression of emotions, problem solving, teaching) scored above baseline. Nurse quality of working life, too, seemed positively impacted. The cultural dimension of nurse quality of working life, that is, the degree to which everyday work activities attune with personal and cultural values, seemed positively impacted, as well, with improvement stable throughout the year following the intervention.

**Conclusions:**

Results support a positive effect of the intervention over both the short term and the medium-to-long term. A brief intervention of the sort may constitute an effective means to improve the nurse-patient relationship by preventing or reducing dehumanising practices.

**Trial registration:**

NCT03283891.

## Background

Patients in the last stage of chronic kidney disease require intensive medical support to prevent death [1]. Kidney transplantation is the solution of choice in this situation, but when this option is not feasible or during the wait time before transplantation, other treatments are needed to support blood purification. The most common of these is haemodialysis (HD), which consists of the extracorporeal removal of waste products from patients’ blood. The treatment must be performed three times a week, generally in a healthcare facility [2].

HD demands a hard commitment from patients in terms of both time management (they must spend three half-days per week in a healthcare facility) and dietary regulation (they are severely limited in the types and quantities of food and liquids that they can consume daily) [3]. Moreover, a broad set of symptoms are associated with HD [4–6]. Whether these have to do with the persistence of the disease or HD side effects, they ultimately have a negative impact on the quality of life (QoL) of patients [7]. Some authors have described the start of HD as a radical transition that leads patients into a new phase of life that requires healthcare professionals to completely reorganise patient care [3]. Given that patients are frequently in HD units for prolonged periods of time, the relationship between patient and healthcare professionals, HD nurses in particular, is critical to patient wellbeing [8, 9]. In this regard, HD nurses must not only provide highly technical treatments but also establish a trusting relationship with patients to create a salutogenic environment that deeply impacts their lifestyle [10, 11]. Without specific training, however, nurses can settle into routine behaviours and lend excessive attention to technical tasks [8]. This can lead to dehumanising practices and a deterioration of the work environment, which in turn can lead to a deterioration of the quality of working life (QWL) of nurses [12]. This situation has frequently been observed and documented in the literature [13–16].

Watson’s Theory of Human Caring [17, 18] is a well-established approach in current nursing practice. Based on humanistic values, this approach encourages nurses to forge a deep and engaged relationship with patients, the ultimate purpose of which is to help patients understand the deep meaning of their existence, suffering, and disharmony [17, 18]. This can be achieved by rooting nursing practice in compassion, active listening, support, reciprocity, and true presence. The core of the transpersonal caring relationship is composed of the following ten dimensions referred to as “carative factors” [17]: (1) forming humanistic-altruistic value systems, (2) instilling faith-hope, (3) cultivating sensibility to self and others, (4) developing a helping-trust relationship, (5) promoting expression of emotions, (6) using problem-solving for decision-making, (7) promoting teaching-learning, (8) promoting a supportive environment, (9) assisting with gratification of human needs, and (10) allowing for existential-phenomenological forces. These factors are often investigated in empirical research because they facilitate operationalisation of the complex concept that is Watson’s transpersonal caring relationship. Empirical instruments to analyse the nurse-patient relationship.

Empirical research has shown that steeping the NPR in Watson’s Theory of Human Caring can be beneficial to both patients and nurses. For patients, it can improve their psychological wellbeing, stress, and QoL; for nurses, it can improve self-confidence, time management, work engagement, and benevolent behaviours (see [19] for a review). Training activities have been developed in the aim of boosting this approach in nursing practice but their efficacy remains poorly documented. In particular, little is known about the efficacy of brief educational activities of the sort included in continuing education programs, normally involving a short-term engagement of no more than a few days or weeks. Pilot studies suggest such activities can be easily incorporated in continuing education programs and improve quality of NPR [19–21]. However, it remains to be seen whether their effects are momentary or lasting. This point is crucial because brief training activities are easy to implement on account of their relatively small cost in terms of both time and money. If found to improve the NPR over the long term, they could serve to prevent acts of dehumanisation and fix problem situations where dehumanising behaviours undermine the quality of the work environment. Against this background, we undertook a study to assess the medium- to long-term effects on caring attitudes and behaviours and on QWL of a brief educational intervention (EI).

## Methods

The EI was tested by way of a cluster randomised controlled trial with repeated measures [22].

### Data collection

Data were collected in the 10 of the 12 hospitals in French-speaking Switzerland with an HD unit, that agreed to take part the study: Geneva (University Hospital, *n* = 18 nurses), Jura (Delémont and Porrentruy site, *n* = 16 nurses), Lausanne (University Hospital, *n* = 11 nurses), Martigny (*n* = 7 nurses), Monthey (*n* = 7 nurses), Morges (*n* = 5 nurses), Payerne (*n* = 7 nurses), Sierre (*n* = 7 nurses), Sion (*n* = 14 nurses), and Yverdon (*n* = 9 nurses). To participate in the study, nurses had to meet the following inclusion criteria: (1) at least 6 months’ HD nursing experience and (2) willingness to participate. There were no exclusion criteria. Data were collected at four time points: immediately prior to the EI (T0), immediately after the EI (T1), 6 months later (T2), and 1 year after the EI (T3). There were 101 nurses at T0, 96 at T1, 86 at T2, and 74 at T3. We first met with management and head nurses of each HD unit to explore their interest in participating in our study. If interested, we next met with nurses to present the project and distribute informed consent forms. Then, we met with the nurses again 2 weeks later to collect the forms. Nurses had the option of mailing them in. A coordinator was appointed for each HD unit to facilitate data collection and communication with researchers. Recruitment took place from April 2016 to January 2017. Our data were naturally structured into 10 clusters (i.e., the ten HD units), as we needed to include the nurses of the same HD unit in either the experimental group (EG) or the control group (CG) to avoid “contamination”. As HD nurses work closely together, splitting those within the unit into the EG and the CG was impossible lest information and practices from the EI leak out of control. Consequently, cluster randomisation was used to define the experimental and control groups. In 2017, prior to data collection, the size of the target population was estimated at 126 HD nurses. We required that both the experimental and the control groups comprise at least 50 nurses. One of the possible cluster combinations that satisfied these conditions was randomly selected using R [23]. The procedure yielded an EG including six clusters (i.e. HD units), for a total of 54 nurses, and a CG including four clusters (i.e. HD units), for a total of 72 nurses. The inclusion process was completed in March 2018 but, owing to the partial withdrawal of one HD unit, the CG shrank considerably. The overall final sample thus totalled 105 HD nurses. Nonetheless, our criterion of at least 50 nurses each in the CG and EG was respected. The final EG comprised 51 nurses and the final CG, 50 (96.2% participation rate in both cases). At each of the four data collection time points, two meetings with nurses were organised: the first to distribute the hardcopy questionnaires and the second to retrieve them. During the first meeting of each time point, nurses were reminded how to correctly complete the self-administered questionnaire. Data thus collected were then transcribed onto digital support. To reduce the risks of introducing transcription errors, data entry was performed twice by different members of the research group and the resulting databases were compared. Up to 1.9% of the values had to be corrected across time points. Because of organisational constraints, researchers were not completely blind to participant group assignment. However, the EI, group assignment, data collection and data analysis were tasks performed for the most part by different members of the research group. Biases were thus kept to a minimum.

### Intervention

Our EI ([24]) consisted of 3.5-h training sessions once a week for 4 weeks. Nurses were organised into groups of no more than five to facilitate interaction and feedback. Theoretical lectures alternated with practical exercises, such as simulations, to ensure the EI fit the reality of each HD unit. At the beginning of each session, focus exercises were proposed to help participants get into a more receptive mind-set and clearly separate the training from previous activities. The intervention was delivered to all nurses in the HD units in the EG by two researchers experienced with the task. The core structure of the intervention was the same to ensure that all of the HD units received the same exposure. However, a certain level of personalisation was required in response to different dynamics of each group.

In the first session, the core concepts of Watson’s [17, 18] Theory of Human Caring were introduced and a first clinical situation was presented to the participants for discussion. In the second session, Watson’s ten carative factors were introduced and discussed. Another clinical situation was presented to show how the concepts covered applied in real life. The third session was dedicated entirely to the concept of “hope” and how to help patients develop it. The concept was presented theoretically and through exercises. Finally, in the fourth session, a simulation activity was organised to revise and practise the concepts covered in the previous sessions. This activity was bookended by a pre-briefing that introduced the situation and helped participants prepare their actions, and a debriefing that served to collect feedback from both participants and instructors. A final evaluation by all the participants wrapped up the training ([24]). For ethical reasons and out of fairness, the EI was offered to the HD units in the CG at study completion.

### Instruments

Data were collected using a three-part questionnaire. The first part served to collect sociodemographic and work-related data. Questions covered gender, age, marital status, and presence of children, prior training in counselling, as well as years of nursing and HD nursing experience. This part of the questionnaire was previously used in related studies ([24]).

The second part consisted of the French version of the Caring Nurse-Patient Interaction Scale (EIIP-70 [25], [26]). The instrument covers 10 dimensions, corresponding to Watson’s 10 carative factors [17]: humanism (6 items), hope (7 items), sensibility (6 items), helping relationship (7 items), expression of emotions (6 items), problem solving (6 items), teaching (11 items), environment (7 items), assistance for basic needs (10 items), and spirituality (6 items). The items refer to nurses’ perceptions of the NPR. They are rated on a five-point Likert scale ranging from “almost never” (1) to “always” (5) to indicate frequency of a given behaviour. This questionnaire had been used before (see the recent review by Cossette [26]) and demonstrated satisfactory psychometric characteristics, Cronbach’s alphas for the 10 dimensions ranged from 0.73 to 0.91 [25]. A pilot study [11] had shown that respondents had no difficulty understanding the questionnaire.

The third part of the questionnaire consisted of the Quality of Work Life Questionnaire by Elizur and Shye [27] translated and validated in French by Delmas, Escobar, and Duquette [4]. This instrument yields a total score that can be divided into four dimensions: psychological (items 1–4), physical (items 5–8), social (items 9–12) and cultural (items 13–16). Respondents must choose from six answers on a Likert scale ranging from 1 “very little” to 6 “a very large part”. Internal consistency was estimated at α = 0.90 for the overall English version [27] and ranged from 0.87 to 0.89 for the dimensions of the French version [4].

### Ethical considerations

Ethical approval was obtained from the Vaud (Switzerland) Ethics Committees on Research Involving Humans (N 2017–00946). Nurses were given 2 weeks’ time to decide whether to participate and to sign the informed consent form. They were free to refuse to participate and to withdraw at any time without consequence. Informed consent was obtained from all participants in the study.

Because of the strong emotions that the EI could elicit, psychological support from a certified psychologist was available for participants at no charge, if needed.

Our research was registered on clinicaltrials.gov (NCT03283891, first registration 14/09/2017).

### Blinding

Given the nature of the intervention, participating nurses could not be blinded to it. As for the research team, data allocation, intervention, data collection and analyses were done by different members of the research team. Nevertheless, no complete blinding was possible for organisational reasons.

### Data analysis

First, means, standard deviations, and frequencies were calculated for sociodemographic and work-related data, as well as data on caring attitudes and behaviours and nurse QWL. Second, EG and CG characteristics were compared at baseline. We used chi-squared tests for categorical variables and linear regressions for numeric variables to test for independence. The Bonferroni correction for *p*-values was applied, given the large number of comparisons made and the fact that these were not part of the core analysis [28]. Third, the EI’s effects on both nurse caring attitudes and nurse QWL were evaluated using random-intercept regression models. This is a particular regression technique that allows the intercept to vary depending on predictors referring to groups of units of analysis instead of the units themselves. This technique is generally used when data are structured in clusters and/or are longitudinal. Our models comprised three levels: observations (regardless of time point), nurses (seen as clusters of observations), and hospitals (seen as clusters of nurses). This model was used to address the problem of lack of independence across observations by the same nurse but at different time points and by nurses working in the same hospital. Statistical significance for all tests was set at *p* < 0.05, following a frequentist approach, and no imputation were used for missing data. All analyses were run on R [23].

## Results

Nurse sociodemographic characteristics are given in Table 1. Across the four time points, the nurses in our sample were predominantly women and had a mean age of about 45 years. Half were married and about three out of four had children. They had about 20 years of nursing experience on average, with more than 12 of these working in HD units. Finally, only one third were previously trained in counselling. No significant differences emerged between the EG and the CG at baseline (T0), confirming that random assignment of participants ensured that potential sources of bias were equally distributed.

**Table 1:** Nurse sociodemographic characteristics by data collection time point

	T0 – pre-intervention	T1 – post intervention	T2–6-month follow-up	T3–12-month follow-up
**Gender, n (%)**				
**Male**	14 (14.1)	12 (12.9)	10 (12.1)	5 (7.0)
**Female**	85 (85.9)	81 (87.1)	73 (87.9)	66 (93.0)
*missing*	*2*	3	3	3
**Marital status, n (%)**				
**Single**	16 (16.1)	15 (16.3)	19 (23.8)	13 (17.6)
**Married**	53 (53.5)	48 (52.2)	44 (55.0)	39 (52.7)
**Widow**	2 (2.0)	2 (2.2)	1 (1.3)	2 (2.7)
**Separated**	10 (10.1)	9 (9.8)	8 (10.0)	6 (8.1)
**De facto**	16 (16.2)	16 (17.3)	5 (6.3)	12 (16.2)
**Civil union**	2 (2.0)	2 (2.2)	3 (3.8)	2 (2.7)
*missing*	*2*	4	6	0
**Children, n (%)**				
**Yes**	74 (74.7)	68 (73.1)	62 (74.7)	54 (76.1)
**No**	25 (25.3)	25 (26.9)	21 (25.3)	17 (23.9)
*missing*	*2*	3	3	3
**Trained in counselling, n (%)**				
**Yes**	33 (33.3)	31 (32.2)	27 (32.5)	24 (33.8)
**No**	66 (66.7)	62 (67.8)	56 (67.5)	47 (66.2)
*missing*	*2*	3	3	3
**Age, years**				
**Mean**	45.3	45.4	46.4	46.5
**SD**	9.8	9.8	9.5	9.4
*missing*	*3*	3	7	6
**Nursing experience, years**				
**Mean**	21.1	21.1	22.5	22.8
**SD**	10.9	10.9	10.3	10.2
*missing*	*3*	3	3	2
**HD nursing experience, years**				
**Mean**	12.1	12.1	13.2	13.1
**SD**	8.3	8.4	8.4	8.3
*missing*	*3*	3	3	2

**Fig. 1:**
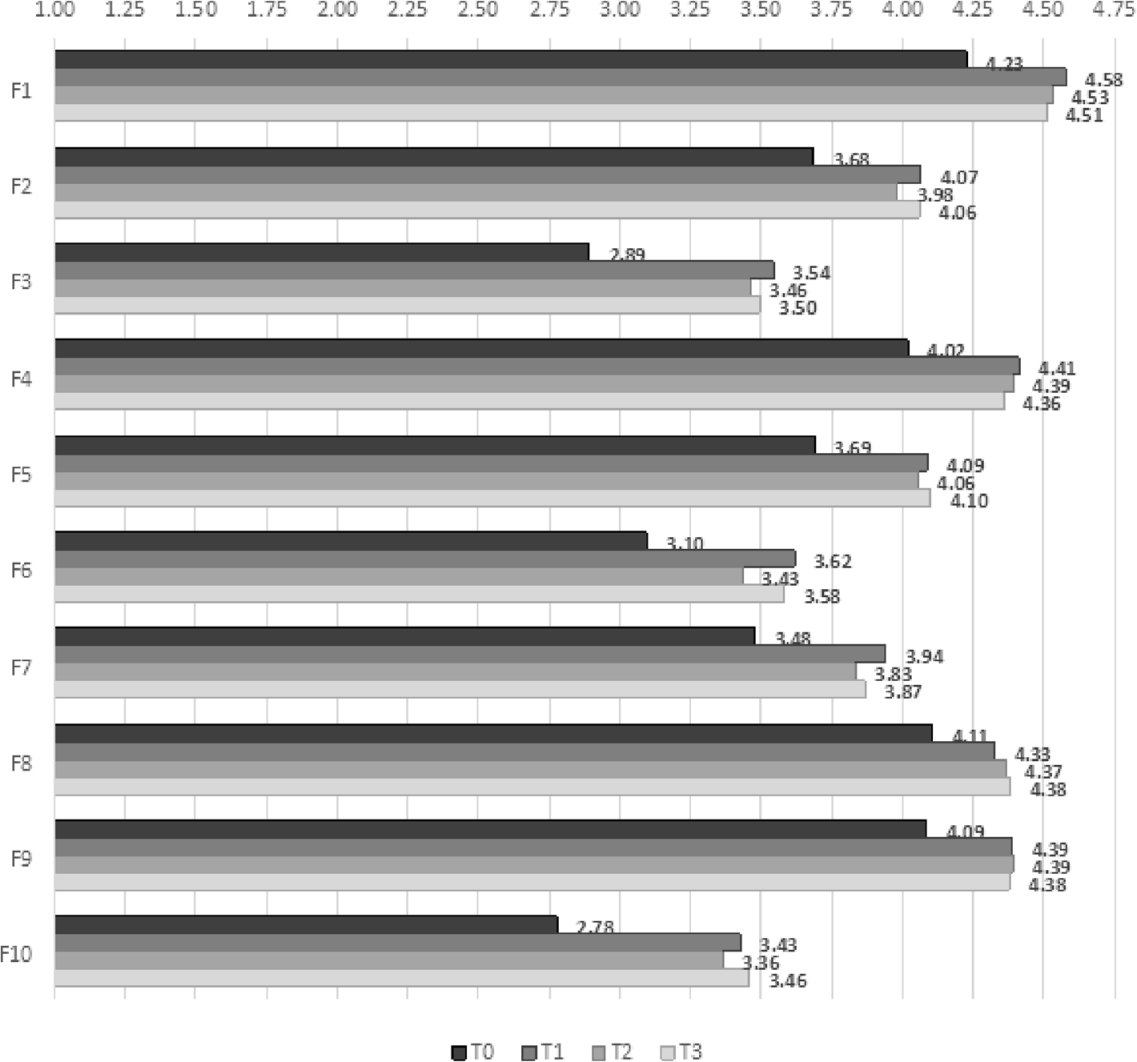
Mean scores for ten carative factors at four data collection time points for experimental group

Regarding the NPR, Fig. 1 shows the mean scores for the EG on the ten dimensions of the EIIP-70 at each time point. There was a noticeable jump across the board from T0 to T1, that is, from pre- to post-intervention. Scores then remained stable 6 months and 1 year later (T2 and T3).

**Fig. 2:**
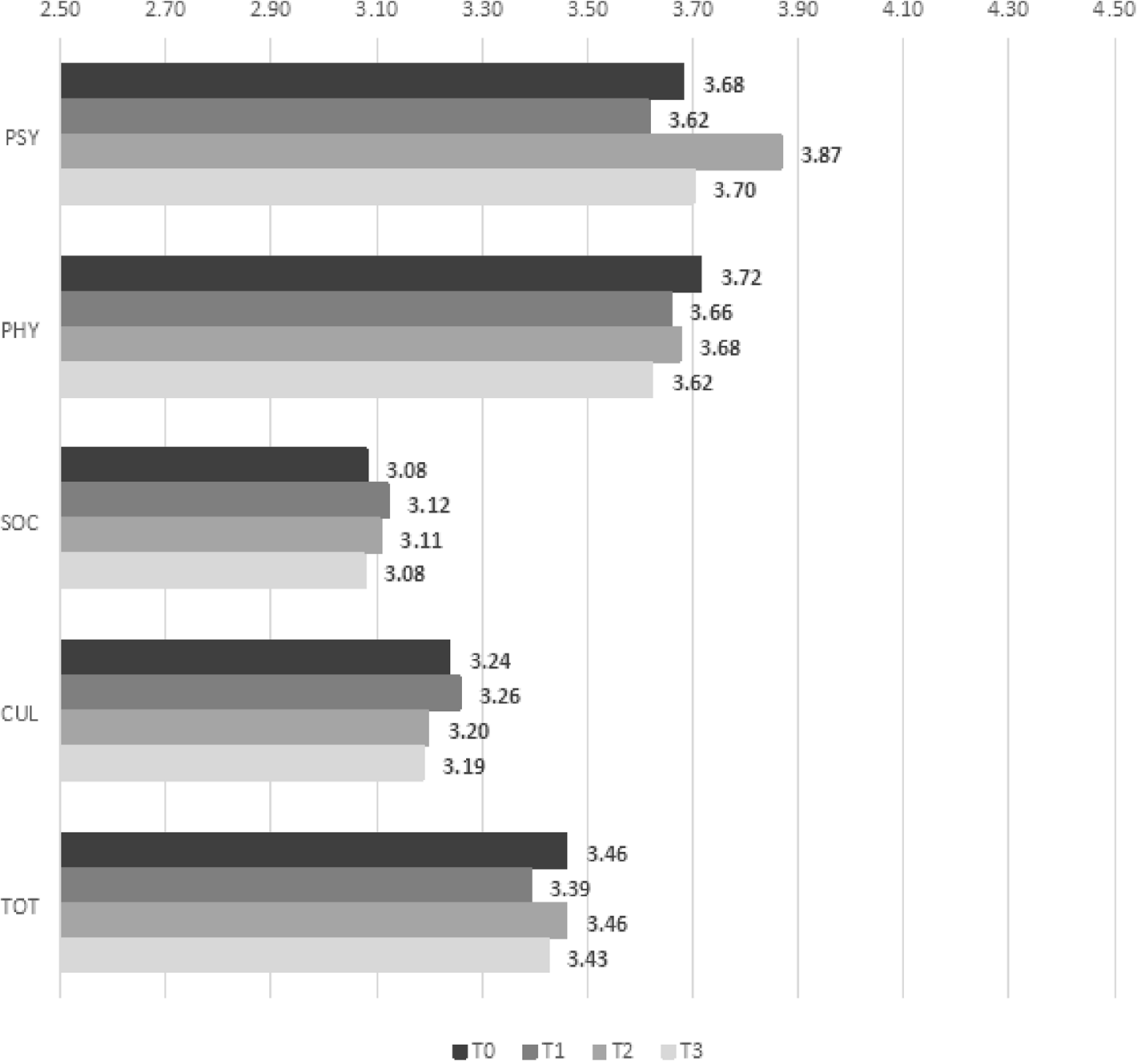
Mean scores for ten carative factors at four data collection time points for control group

Figure 2 shows that, meanwhile, the mean scores for the CG changed only slightly over time and only on the following four dimensions: *F2-Hope*, *F3-Sensibility*, *F6-Problem Solving*, and *F10-Spirituality*.

We used multilevel regression analysis to evaluate the differences between the trends in the EG and the CG. Four main patterns emerged (Table 2). Against the trend in the CG used as baseline, five factors obtained significantly higher scores at T1 than at T0: *F1-Humanism* (β = 0.30, *p* = 0.01), *F3-Sensibility* (β = 0.34, *p* = 0.02), *F4-Helping Relationship* (β = 0.21, *p* = 0.03), *F7-Teaching* (β = 0.30, *p* = 0.03), and *F9****-****Needs* (β = 0.27, *p* = 0.01). Scores then decreased slightly over time. The difference with T0 remained significant for three factors at T2 and T3: *F3-Sensibility* (T2: β = 0.40, *p* = 0.01; T3: β = 0.38, *p* = 0.02), *F4-Helping Relationship* (T2: β = 0.26, *p* = 0.01; T3: β = 0.28, *p* = 0.01), and *F7-Teaching* (T2: β = 0.49, *p* < 0.01; T3: β = 0.43, *p* < 0.01). For the other two factors, the difference with T0 remained statistically significant only at T2: *F1-Humanism* (T2: β = 0.37, *p* < 0.01; T3: β = 0.22, *p* = 0.07) and *F9-Needs* (T2: β = 0.29, *p* = 0.01; T3: β = 0.19, *p* = 0.11).

**Table 2:** Differences in change in caring factors evaluated via random-intercept regression models

		Beta	***P***			Beta	***P***
**F1 - Humanism**	**T1-T0**	0.30	0.01	**F6 - Problem**	**T1-T0**	0.25	0.12
	**T2-T0**	0.37	< 0.01	**Solving**	**T2-T0**	0.35	0.04
	**T3-T0**	0.22	0.07		**T3-T0**	0.44	0.01
	**T2-T1**	0.07	0.71		**T2-T1**	0.10	0.72
	**T3-T1**	−0.08	0.25		**T3-T1**	0.19	0.86
	**T2-T3**	− 0.15	0.12		**T2-T3**	0.09	0.70
	**N (level 1)**		357		**N (level 1)**		351
	**N (level 2)**		101		**N (level 2)**		99
	**N (level 3)**		10		**N (level 3)**		10
	**Variance of level 2**		0.07		**Variance of level 2**		0.34
	**Variance of level 3**		< 0.01		**Variance of level 3**		< 0.01
	**Variance of residual**		0.15		**Variance of residual**		0.3
**F2 - Hope**	**T1-T0**	0.20	0.12	**F7 - Teaching**	**T1-T0**	0.30	0.03
	**T2-T0**	0.23	0.08		**T2-T0**	0.49	< 0.01
	**T3-T0**	0.31	0.03		**T3-T0**	0.43	< 0.01
	**T2-T1**	0.03	0.59		**T2-T1**	0.19	0.91
	**T3-T1**	0.11	0.78		**T3-T1**	0.13	0.80
	**T2-T3**	0.08	0.71		**T2-T3**	−0.06	0.34
	**N (level 1)**		355		**N (level 1)**		349
	**N (level 2)**		100		**N (level 2)**		99
	**N (level 3)**		10		**N (level 3)**		10
	**Variance of level 2**		0.26		**Variance of level 2**		0.24
	**Variance of level 3**		< 0.01		**Variance of level 3**		< 0.01
	**Variance of residual**		0.19		**Variance of residual**		0.22
**F3 - Sensibility**	**T1-T0**	0.34	0.02	**F8 -**	**T1-T0**	0.11	0.27
	**T2-T0**	0.40	0.01	**Environment**	**T2-T0**	0.23	0.03
	**T3-T0**	0.38	0.02		**T3-T0**	0.16	0.16
	**T2-T1**	0.06	0.65		**T2-T1**	0.12	0.87
	**T3-T1**	0.04	0.60		**T3-T1**	0.05	0.66
	**T2-T3**	−0.02	0.45		**T2-T3**	−0.07	0.26
	**N (level 1)**		351		**N (level 1)**		354
	**N (level 2)**		98		**N (level 2)**		100
	**N (level 3)**		10		**N (level 3)**		10
	**Variance of level 2**		0.3		**Variance of level 2**		0.11
	**Variance of level 3**		< 0.01		**Variance of level 3**		< 0.01
	**Variance of residual**		0.26		**Variance of residual**		0.13
**F4 - Helping**	**T1-T0**	0.21	0.03	**F9 - Needs**	**T1-T0**	0.27	0.01
**Relationship**	**T2-T0**	0.26	0.01		**T2-T0**	0.29	0.01
	**T3-T0**	0.28	0.01		**T3-T0**	0.19	0.11
	**T2-T1**	0.05	0.68		**T2-T1**	0.02	0.57
	**T3-T1**	0.06	0.73		**T3-T1**	−0.09	0.22
	**T2-T3**	0.02	0.56		**T2-T3**	−0.11	0.18
	**N (level 1)**		354		**N (level 1)**		355
	**N (level 2)**		100		**N (level 2)**		99
	**N (level 3)**		10		**N (level 3)**		10
	**Variance of level 2**		0.15		**Variance of level 2**		0.12
	**Variance of level 3**		0.01		**Variance of level 3**		< 0.01
	**Variance of residual**		0.11		**Variance of residual**		0.13
**F5 - Expression of**	**T1-T0**	0.18	0.14	**F10 -**	**T1-T0**	0.25	0.17
**Emotions**	**T2-T0**	0.31	0.02	**Spirituality**	**T2-T0**	0.39	0.04
	**T3-T0**	0.37	0.01		**T3-T0**	0.33	0.10
	**T2-T1**	0.12	0.83		**T2-T1**	0.14	0.77
	**T3-T1**	0.19	0.91		**T3-T1**	0.08	0.65
	**T2-T3**	0.06	0.67		**T2-T3**	−0.06	0.38
	**N (level 1)**		354		**N (level 1)**		339
	**N (level 2)**		101		**N (level 2)**		91
	**N (level 3)**		10		**N (level 3)**		10
	**Variance of level 2**		0.20		**Variance of level 2**		0.57
	**Variance of level 3**		0.01		**Variance of level 3**		0.01
	**Variance of residual**		0.18		**Variance of residual**		0.37

Second, two factors obtained significantly higher scores only as of T2: *F5-Expression of Emotions* (T2: β = 0.31, *p* = 0.02; T3: β = 0.37, *p* = 0.01) and *F6-Problem Solving* (T2: β = 0.35, *p* = 0.04; T3: β = 0.44, *p* = 0.01). Third, *F2-Hope* obtained a significantly higher score only at T3 (T3: β = 0.31, *p* = 0.03). Fourth, *F8-Environment* and *F10-Spirituality* obtained significantly higher scores only at T2 (respectively, β = 0.23, *p* = 0.03, and β = 0.39, *p* = 0.04).

**Fig. 3:**
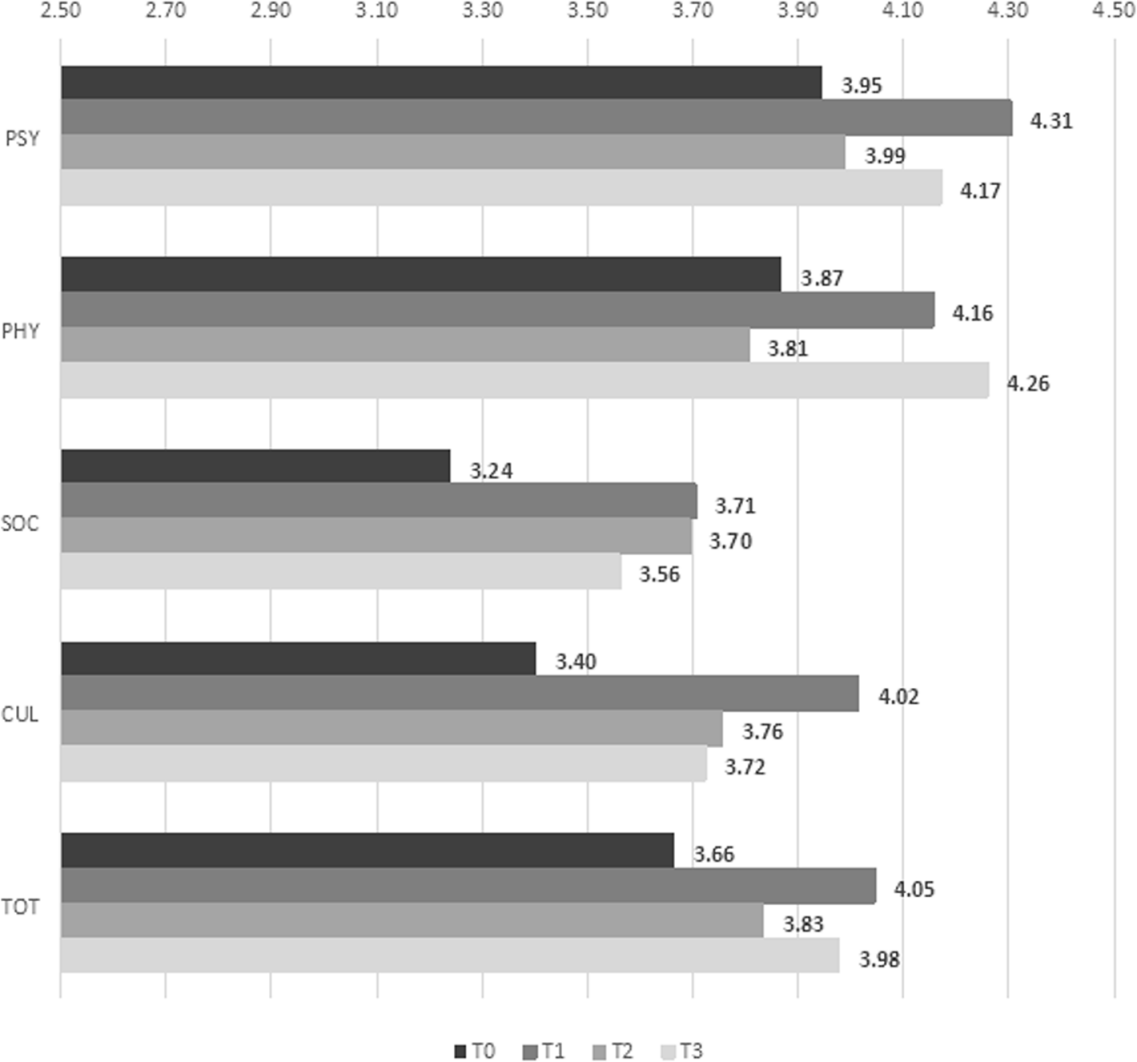
Quality of working life dimensions by data collection time point for experimental group

Figure 3 illustrates change over time in nurse QWL for the EG. The mean scores obtained across the data collection time points were quite erratic. Those for the physical and psychological dimensions increased immediately post-intervention (T1) but the statistically significant change disappeared at T2 only to reappear at T3. A similar trend was observed for the cultural dimension, where scores increased at T1 but decreased less sharply at T2, remaining above the baseline score. Finally, the social dimension showed a unique trend in that scores increased post-intervention (T1) and remained stable over time.

**Fig. 4:**
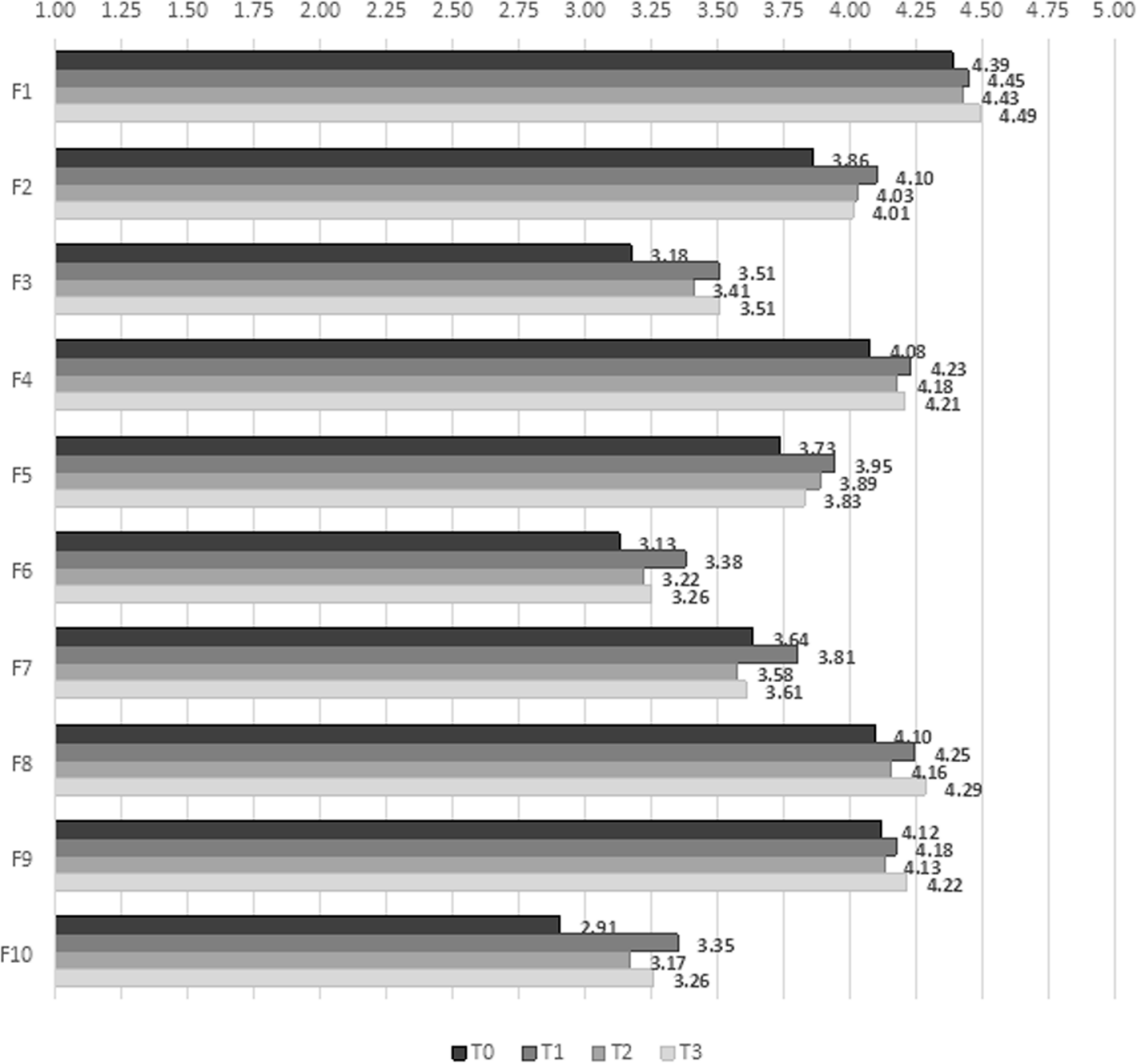
Quality of working life dimensions by data collection time point for control group

Where the CG is concerned, Fig. 4 shows that there was no significant change over time for any of the dimensions.

Regression models (Table 3) were used to examine the differences between the trends in the EG and the CG. Against the trend in the CG used as baseline, results showed that the EG obtained significantly higher scores only on the psychological and the cultural dimensions immediately post-intervention (respectively, β = 0.48, *p* = 0.01, and β = 0.69, *p* < 0.01). The difference persisted over time only for the cultural dimension (T2: β = 0.46, *p* = 0.04; T3: β = 0.51, *p* = 0.03). Instead, a sharp decrement at T2 (T2-T1: β = − 0.49, *p* = 0.01) brought the values for the psychological dimension back to baseline. A significant increment was observed also for total QWL at T1 (β = 0.41, *p* = 0.03), but this did not last over time.

**Table 3:** Differences in change in QWL dimensions evaluated via random-intercept regression models

		Beta	***P***			Beta	P
**Psychological QWL**	**T1-T0**	0.48	0.01	**Social QWL**	**T1-T0**	0.39	0.13
	**T2-T0**	−0.02	0.93		**T2-T0**	0.21	0.42
	**T3-T0**	0.13	0.53		**T3-T0**	0.17	0.53
	**T2-T1**	−0.49	0.01		**T2-T1**	−0.18	0.24
	**T3-T1**	−0.34	0.05		**T3-T1**	−0.22	0.21
	**T2-T3**	0.15	0.76		**T2-T3**	−0.04	0.44
	**N (level 1)**		340		**N (level 1)**		330
	**N (level 2)**		92		**N (level 2)**		86
	**N (level 3)**		10		**N (level 3)**		10
	**Variance of level 2**		0.51		**Variance of level 2**		0.71
	**Variance of level 3**		0.05		**Variance of level 3**		< 0.01
	**Variance of residual**		0.42		**Variance of residual**		0.67
**Physical QWL**	**T1-T0**	0.38	0.11	**Cultural QWL**	**T1-T0**	0.69	< 0.01
	**T2-T0**	0.02	0.94		**T2-T0**	0.46	0.04
	**T3-T0**	0.44	0.08		**T3-T0**	0.51	0.03
	**T2-T1**	−0.36	0.07		**T2-T1**	−0.23	0.15
	**T3-T1**	0.06	0.60		**T3-T1**	−0.18	0.22
	**T2-T3**	0.42	0.95		**T2-T3**	0.05	0.59
	**N (level 1)**		340		**N (level 1)**		332
	**N (level 2)**		95		**N (level 2)**		86
	**N (level 3)**		10		**N (level 3)**		10
	**Variance of level 2**		0.49		**Variance of level 2**		0.68
	**Variance of level 3**		0.07		**Variance of level 3**		< 0.01
	**Variance of residual**		0.63		**Variance of residual**		0.51
**Total QWL**	**T1-T0**	0.41	0.03				
	**T2-T0**	0.15	0.42				
	**T3-T0**	0.30	0.12				
	**T2-T1**	−0.26	0.08				
	**T3-T1**	−0.10	0.29				
	**T2-T3**	0.15	0.79				
	**N (level 1)**		318				
	**N (level 2)**		78				
	**N (level 3)**		10				
	**Variance of level 2**		0.56				
	**Variance of level 3**		0.01				
	**Variance of residual**		0.32				

## Discussion

The main objective of our research was to determine whether a brief EI based on Watson’s Theory of Human Caring [17, 18] had lasting effects on the NPR and on QWL for HD nurses. According to a recent review [26], such interventions had only ever been evaluated in pilot studies with small samples [19–21] and effects over the medium-to-long term were never explored. To our knowledge, then, our study is the first to test a brief EI for nurses and to report reliable estimates of its effects immediately post-intervention and of changes to these over the following year.

Regarding the NPR, our results show that study participants reported medium-to-high levels of caring attitudes and behaviours already at pre-intervention. Under the circumstances, the little room for improvement made it difficult for any intervention to have a strong impact. Still, the results show that our EI had a clear effect on nurse caring attitudes and behaviours. Five of the ten carative factors showed an improvement immediately post-intervention (T1), in line with what has been observed in other studies [19–21]. In the year following the EI, that is, across the three post-intervention time points, we observed that each of the ten carative factors was positively impacted by the EI at least once at some point. This shows that the EI touched on and affected all aspects of the NPR, though more time was needed for some effects to surface.

In fact, the longitudinal perspective of our study shows that the EI has a strong effect on some carative factors that can be observed immediately post-intervention (T1) and that it persists over time. This was the case for *F3-Sensibility*, *F4-Helping Relationship*, *F7-Teaching* and, arguably, *F1-Humanism* and *F9-Needs.* For other factors, the intervention had a delayed effect. This was the case for *F5-Expression of Emotions* and *F6-Problem Solving*, which registered a change only later at six-month follow-up (T2) and maintained it at one-year follow-up (T3), and maybe for *F2-Hope*, which registered a statistically significant change only at one-year follow-up (T3). All these factors describe elements that are crucial for nursing and represent an added value of the profession.

The results regarding the two last carative factors, *F8-Environment* and *F10-Spirituality,* are more peculiar in that as they follow an erratic pattern. The trend clearly showed that the EI had a positive effect but differences between the EG and the CG were significant only at T2. The reason for this cannot be fully understood without further qualitative research. However, we can say that these two carative factors have something in common: they describe elements that are not limited to the spheres of nurse attitudes and nurse-patient interactions. *F8-Environment* concerns the creation of a supportive, protective, and caring environment. This is something that depends not only on nurse attitudes and actions but also on how HD units are organised, both formally and informally. *F10-Spirituality* refers to the inclusion of spiritual and existential beliefs in the healthcare routines. Despite being deeply personal, these beliefs are also deeply rooted in cultural schemas that suggest how these issues must be addressed and by whom. In both cases, nurses are in a delicate situation of having to act with strong cultural and organisational boundaries. As widely discussed in the field of social science (for different points of view see [29–32]), individual agency is not completely free. It must be contextualized within the structural limitations of the social framework in which people function. This “agency within a structure” ([30], p. 41) implies that there are limits that individual action cannot overcome without being socially sanctioned. These limits can be explicit, such as written regulations, but also implicit, such as traditional or routine behaviours. In the context of HD units, many factors, independent of the will of nurses, may affect the quality of the work environment and limit their possibilities for change. Roch et al. [33] demonstrated that the organizational climate affected nurse caring behaviour. Poor relations with management, understaffing, and inadequate structures are but three factors completely outside the control of nurses that may work against them and afford little room to shape the environment according to the principles of caring. The EI targeted the attitudes and behaviours of nurses working in HD units. Consequently, it is not surprising that it had little impact on the general work organisation of these settings. The same could be said about *F10-Spirituality* and why there was no consequent shift of individual values and spiritual/existential beliefs towards a more humanistic perspective. Individual spiritual life is framed within a social context that goes far beyond nurse-patient interaction. Though working in HD units places nurses in contact with death and suffering, elements usually central to the development of spirituality, the role of religious institutions and spiritual traditions cannot be readily dismissed when describing the reshaping of individual beliefs. Moreover, unlike in North America, where both the theoretical model and the instrument of measure that we used were developed, in Switzerland religious and spiritual matters are rarely discussed in the public sphere. This specific cultural context probably creates further difficulties for both nurses and patients that prevent any enduring change in this regard.

In the light of these results, it is safe to say that the EI falls short of having a lasting effect on factors that entail a marked collective dimension. Adapting our intervention and empirical instruments to some degree to the local cultural and organisational situations may be a path for further research. Perhaps, however, there is no possible solution. After all, the EI targets the NPR and, as far as the factors that relate directly to it or to the inner attitudes of nurses when functioning within this context are concerned, we did observe changes that persisted over time. Instead, when larger social structures are involved, the effect seems not to stick. This is consistent with the objectives of the EI, which is not meant to have a broad effect but rather to reshape a specific relationship crucial to both nurse and patient.

The second expected effect of the EI was on nurse QWL. Unlike the effect on the NPR, this was to be an indirect effect, given that the focus of the EI was on rooting the NPR in the principles of human caring. A change in nurse QWL was expected as a consequence of changes in the NPR.

Two dimensions of nurse QWL were clearly affected by the EI, but in different ways. On the one hand, the psychological dimension registered a sharp increase right after the EI (T1). However, this was erased by a sharp decline 6 months later (T2). Based on the data collected, we cannot specify the dynamics behind this and explain why the initial effect did not persist. However, the simplest and perhaps most realistic hypothesis for returning to baseline levels is that the immediate effects of the EI upon completion, which evidently had repercussions on the psychological status of nurses, rapidly faded once the initial enthusiasm generated by the intervention waned.

On the other hand, the cultural dimension of nurse QWL, that is, how everyday work activities are in line with personal values and cultural beliefs, increased after the intervention and remained stable above baseline. This suggests that the EI narrowed an existing gap between how nurses conceive the best way to practise their profession and what they actually do in their everyday reality. Though further research is needed to support this finding, it suggests that Watson’s Theory of Caring is in line with nurses’ expectations and desires regarding the profession and that this might help counter detrimental phenomena, such as absenteeism and professional dropout.

Finally, the two remaining dimensions of nurse QWL, that is, the physical and the social dimensions, were not impacted by the EI. However, this “non-result” paradoxically supports the efficacy of our EI. The physical dimension of nurse QWL refers to physical health and the influence that work has on it, whereas the social dimension refers to whether nurses feel that their everyday work activities have a positive effect on society at large. As these elements were disconnected from the objectives of our EI, the absence of noticeable change in this regard supports the idea that the EI helps develop specific attitudes and behaviours and does not simply have a general placebo effect that improves all and any indicator considered. This is in line with previous literature [19] that suggests that different EI lead to different outcomes. Consequently, it is important for the goals of each EI to be defined in advance and adapted to each context.

### Limitations

Our study has three limitations that need to be addressed. First, due to organisational constraints, some members of the research group were involved in all phases of the research. Consequently, the blinding process was not perfect. Second, unsurprisingly for a panel study, our sample suffered 26.7% attrition from T0 to T3. Even if this sample reduction occurred mostly between T2 and T3, a selection bias may be present in the analysis, due to a non-random sample reduction. Third, overall levels of caring behaviours and attitudes may be overestimated owing to social desirability and the Hawthorne effect. However, this would affect only the descriptive analyses. The main analyses are not affected by this thanks to the randomised controlled trial design.

## Conclusion

For the first time ever to our knowledge, an experimental design was applied to a large sample of nurses working in HD units to test the effects on the NPR and on nurse QWL of a brief EI based on Watson’s Theory of Human Caring [17, 18].

Our study supports previous evidence from pilot studies of the efficacy of some brief interventions in giving nurse practices and behaviours a more humanistic orientation [19–21]. These effects appear stable at least through the year following the intervention. Moreover, our results suggest that this shift in practice may improve nurse QWL by closing the gap between what nurses believe nursing should be and how they experience it in their everyday reality.

Given these results, brief EI, such as the one studied [21], appear to be an effective means of improving the NPR by preventing dehumanising practices or reducing them if already present. Brief interventions are easy to incorporate in continuing professional development programs and, consequently, could be a time- and cost-efficient means of improving the NPR and, ultimately, nurse QWL.

## Data Availability

The datasets generated and/or analysed during the current study are not publicly available owing to privacy regulations and the agreement between researchers and participants but are available from the corresponding author on reasonable request.
